# Circular RNAs in Intestinal Mucosal Homeostasis and Pathologies

**DOI:** 10.3390/ncrna12030014

**Published:** 2026-04-30

**Authors:** Hee Kyoung Chung, Lan Xiao, Jian-Ying Wang

**Affiliations:** 1 Cell Biology Group, Department of Surgery, University of Maryland School of Medicine, Baltimore, MD 21201, USA; hkchung@som.umaryland.edu (H.K.C.); lxiao@som.umaryland.edu (L.X.); 2 Baltimore Veterans Affairs Medical Center, Baltimore, MD 21201, USA; 3 Department of Pathology, University of Maryland School of Medicine, Baltimore, MD 21201, USA

**Keywords:** epithelium integrity, mucosal injury and repair, RNA-binding proteins, long noncoding RNAs, IBD

## Abstract

The mammalian intestinal epithelium is a rapid self-renewal tissue in the body, serving as a critical interface between the host and the external environment. Maintaining the intestinal epithelium homeostasis requires precise coordination of cellular processes, including proliferation, migration, differentiation, autophagy, and cell-to-cell interaction. An increasing body of evidence has unveiled circular RNAs (circRNAs) as abundant and stable regulatory molecules that play pivotal roles in the intestinal epithelial biology and are intimately involved in many aspects of gut mucosal pathologies. Unlike linear RNAs, circRNAs form covalently closed loop structures through back-splicing events, conferring remarkable stability and resistance to exonucleolytic degradation. circRNAs regulate the growth of the intestinal mucosa, injury-induced epithelial regeneration, and gut barrier function via diverse mechanisms, including interactions with microRNAs and RNA-binding proteins. Deregulated circRNAs are implicated in the pathogenesis of various gut mucosal disorders such as inflammatory bowel disease and malignancies. In this review, we highlight pathobiological functions and mechanisms of intestinal epithelium-enriched circRNAs, particularly *circHIPK3*, *Cdrlas*, and *circPABPN1*, in the epithelium homeostasis and pathologies and also discuss potential clinical application of circRNAs as diagnostic biomarkers and therapeutic targets in patients with critical diseases.

## 1. Introduction

The intestinal epithelium serves as a critical biological barrier, simultaneously facilitating nutrient absorption while defending against luminal pathogens, toxins, and antigens. This single layer of columnar cells lining the luminal surface of the intestinal mucosa is continuously renewed, with the average epithelial cell lasting only 5–7 days in humans [1,2]. Impaired integrity of the intestinal epithelium occurs commonly in various pathologies and contributes to numerous gut disorders, including inflammatory bowel disease (IBD), infectious enterocolitis, colorectal cancer, and critical surgical disorders, but the exact mechanisms underlying intestinal mucosal homeostasis and disruption in various pathophysiological conditions remain largely unknown.

Over the past decade, circular RNAs (circRNAs) have emerged as important regulatory molecules in intestinal epithelial pathophysiology. Initially dismissed as rare splicing aberrations when first observed in the 1970s in viroids [3,4], circRNAs are now recognized as abundant, evolutionarily conserved molecules and are dynamically expressed across different mammalian tissues and developmental stages [5,6,7]. High-throughput RNA sequencing and computational algorithms designed to detect back-splice junctions have revealed thousands of circRNAs in the intestinal epithelium. While this review focuses on the biogenesis and physiological functions of circRNAs, it is important to note that their identification via sequencing is routinely validated through orthogonal methods. These include RT-qPCR using divergent primers to amplify back-splice junctions, resistance to RNase R digestion, and confocal microscopy (such as RNA-FISH) to confirm precise subcellular localization [8].

CircRNAs exhibit exclusive characteristics that distinguish them from linear RNAs, and their covalently closed circular structure renders them highly resistant to exonuclease degradation, resulting in remarkable stability and extended half-lives. Many circRNAs have tissue-specific expression patterns, with certain circRNAs accumulating to levels exceeding their linear cognate mRNAs [9,10]. The mucosal tissues of the gastrointestinal (GI) tract display particularly rich circRNA expression profiles, with thousands of circRNAs that are identified in specific epithelial cells and expressed differentially in various mucosal pathological states [11]. Although some circular transcripts have been identified as templates for cap-independent translation into bioactive peptides, the present review focuses specifically on the noncoding regulatory networks that govern intestinal mucosal homeostasis.

Functional studies have revealed that specific circRNAs modulate intestinal mucosal growth, epithelial barrier function, autophagy, inflammatory responses, and epithelial repair after injury. *CircPABPN1* protects against intestinal injury through regulation of autophagy [12], whereas *circHIPK3* regulates epithelial cell proliferation and mucosal repair [13]. Recently, circRNA *Cdr1as* has been identified as a biological regulator affecting intestinal mucosal renewal and injury-induced epithelial regeneration [14]. This review provides a comprehensive overview of circRNAs in maintaining the intestinal epithelium homeostasis by altering epithelial renewal, mucosal repair after injury, and gut barrier function. We also highlight the molecular mechanisms by which circRNAs modulate gene expression via interaction with microRNAs (miRNAs) and RNA-binding proteins (RBPs) and point out the involvement of altered circRNAs in the pathogenesis of human gut mucosal disorders.

## 2. Biogenesis of Circular RNAs

### 2.1. Definition and Structural Features of circRNAs

**Figure 1:**
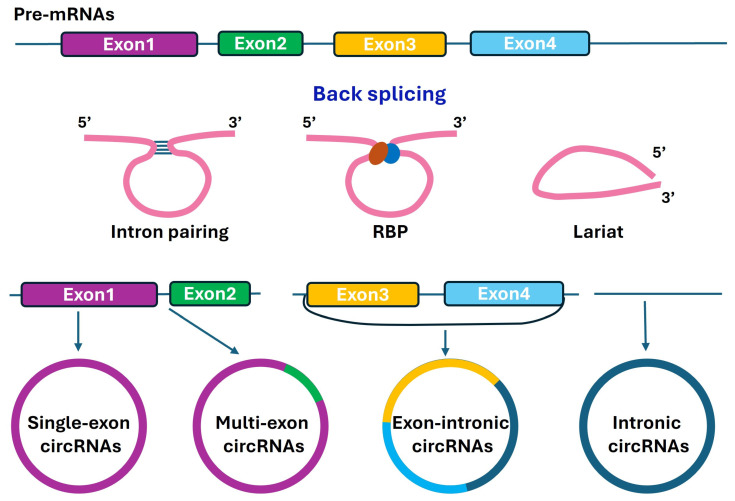
A schematic of circRNA biogenesis. CircRNAs are formed through back-splicing during pre-mRNA processing by the spliceosome, leading to an RNA molecule in the circular conformation with a 3′−5′ phosphodiester bond at the back-splicing junction site. Since back-splicing is inefficiently processed, the basal expression levels of circRNAs are relatively low in most cells and tissues in general.

The intestinal epithelium expresses a large number of circRNAs that represent a special class of widespread and diverse endogenous long noncoding RNAs. CircRNAs are single-stranded RNA molecules characterized by their covalently closed circular structure, lacking the five-prime cap and three-prime poly-A tail that define conventional linear mRNAs [15,16]. This unique topology arises from back-splicing, in which a downstream splice donor joins to an upstream splice acceptor, creating a continuous loop with no free ends. Based on genomic origin, circRNAs are often grouped into three major categories [17,18]: (1) single- or multi-exon circRNAs that are the most abundant type, consisting entirely of exonic sequences and typically residing in the cytoplasm where they interact with target miRNAs and RBPs; (2) intronic circRNAs that are composed of intronic sequences and escape debranching, remaining predominantly in the nucleus where they regulate gene transcription; and (3) exon–intron circRNAs that contain both exonic and intronic sequences (Figure 1). Although circRNA size varies considerably, ranging from less than 100 nucleotides to over 4 kilobases, most circRNAs fall within 200–800 nucleotides [19].

### 2.2. Biogenesis Pathways and Cellular Localization

CircRNA biogenesis occurs through back-splicing during pre-mRNA processing in the mammal tissues [20,21]. Intron-pairing driven circularization is the most well-characterized mechanism. Complementary sequences in flanking introns, such as inverted Alu repeats in mammalian genomes, base-pair to form stem–loop structures that bring splice sites together, favoring back-splicing over canonical forward splicing, while RBP-mediated circularization provides an alternative mechanism [22,23]. Specific RBPs such as quaking can bind to motifs in flanking introns and function through protein–protein interactions, bridging distant splice sites [24,25].

The lack of 5′ and 3′ ends protects circRNAs from exonuclease-mediated decay, significantly increasing their half-life compared to linear isoforms [6,26,27]. This enhanced stability allows circRNAs to accumulate to high steady-state levels in cells despite potentially low transcription rates. CircRNA degradation occurs through endonucleolytic cleavage, with RNA-binding proteins, including AGO2 and RNase L, implicated under specific conditions [28]. N6-methyladenosine modifications also regulate stability [12]. Most exonic circRNAs are exported to the cytoplasm, where they interact with miRNAs, RBPs, and ribosomes. Nuclear circRNAs are retained through mechanisms involving intronic sequences and often localize to transcription sites [18].

## 3. CircRNAs in the Intestinal Epithelium Homeostasis

Emerging evidence indicates that circRNAs function as master regulators of intestinal mucosal homeostasis and that control of the levels of cellular circRNAs is crucial for maintaining the epithelium integrity under physiological and pathological conditions [13]. Table 1 provides a list of selected circRNAs that have in vivo and in vitro experimental evidence to either support a stimulatory, suppressive, or context-dependent role in controlling the intestinal mucosal growth, injury-induced epithelial regeneration, epithelium-host defense, and gut barrier function. Many of these studies involve the generation of intestinal epithelial tissue-specific knockout mouse models and the delivery and/or transfection of circRNA transgenes or specific small interfering RNAs (siRNAs) targeting given circRNAs that alter cellular circRNA levels. Table 1 further provides instances of the implication of circRNAs in gut mucosal disorders and the mechanisms underlying circRNAs in the regulation of intestinal mucosal adaptation.

### 3.1. circHIPK3

Genome-wide profile analyses have been performed in the small intestinal epithelium from a mouse septic model induced by cecal ligation and puncture (CLP) to determine the involvement of circRNAs in the gut epithelium homeostasis [13]. Exposure of mice to CLP for 48 h induces mucosal lesions in the small intestine, as indicated by edematous and swollen mucosa with areas of red streaks macroscopically, and by severely sloughed cells, denuded villi with dilated capillaries, and frank hemorrhage microscopically [48,49,50,51]. CLP for 48 h also results in an acute gut barrier dysfunction, as evidenced by an increased gut permeability to fluorescein isothiocyanate (FITC) dextran [48,49]. In this mucosal injury model, total RNA was isolated from the small intestinal mucosal tissues and then digested with RNase R to remove all linear RNAs. The remaining circRNAs were amplified and transcribed into fluorescent cRNA utilizing a random priming method, and microarray-based interrogation of global circRNA expression profiles was carried out. In total, ~9360 circRNAs are detectable in the small intestinal mucosa of the mouse.

A comparison of the circRNA expression profiles in control mice relative to mice exposed to CLP for 48 h reveals that ~300 circRNAs, including *circHIPK3*, are differentially expressed in the damaged mucosa. Although some circRNAs that showed significant changes in CLP-mice are undetectable in human intestinal epithelial cells (IECs), the basal level of *circHIPK3* is higher than that of many other circRNAs detected in the mouse intestinal mucosal tissue and in cultured human and rat IECs. *CircHIPK3* is transcribed from the exon 2 of the *HIPK3* gene and generated by direct back-splicing supported by intronic RNA pairings [29,52]. *CircHIPK3* is expressed in various mammalian tissues and has cell-type-specific regulation of cell functions by interacting with different miRNAs [53]. Expression levels of *circHIPK3* change remarkably in pathological conditions such as cancer, but it can be tumor-repressive or pro-oncogenic, depending on its cellular context, target miRNAs, and tumor type [29,53].

*CircHIPK3* regulates intestinal epithelial repair in an in vitro epithelial injury model, since elevation of *circHIPK3* abundance enhances intestinal epithelial repair after wounding [13]. In the control group, epithelial repair occurs quickly, as demonstrated by a significant increase in cells migrating over the denuded (wounded) area at 6 h; the wounded areas are further healed at 16 and 24 h thereafter [54,55]. Ectopically expressed *circHIPK3* stimulates epithelial repair after wounding, and the number of cells over the denuded area is much higher in cells overexpressing *circHIPK3* than in cells transfected with a control vector. In contrast, decreasing the levels of endogenous *circHIPK3* by transfection with siRNA specifically targeting the circularization junction of *circHIPK3* inhibits intestinal epithelial repair after wounding. Experiments examining expression patterns of cell migration- and proliferation-associated proteins show that the levels of small Rho GTPases Rac1, Cdc42, and RhoA, and cell division-related protein cyclin B1 increase rapidly in the control group after wounding, but this induction is abolished by *circHIPK3* silencing. Consistent with a reduction in these epithelial repair-associated factors, *circHIPK3* silencing also decreases actin dynamics in migrating cells at early epithelial restitution. Significant actin-based plasma membrane protrusions, including membrane ruffles, blebs, and lamellipodia, at the wounding edge occur at 3 and 6 h after epithelial injury in control cells, which is prevented by *circHIPK3* silencing. These findings indicate that *circHIPK3* affects intestinal epithelial repair at least in part by altering the activity of Rac1, Cdc42, and RhoA and by modulating the expression levels of cyclin B1.

*CircHIPK3* enhances the renewal of the intestinal epithelium. In cultured human IECs in vitro, the decrease in *circHIPK3* levels lowers proliferation, as shown by a significant decrease in cell numbers [13]. In primarily cultured intestinal organoids derived from mouse intestinal crypts, decreasing *circHIPK3* levels also inhibits the mucosal growth ex vivo, since the numbers of BrdU-positive cells and the sizes of intestinal organoids decrease significantly in the *circHIPK3*-deficient group relative to controls. *CircHIPK3* silencing also decreases the levels of Cdc42, Rac1, RhoA, and cyclin B1 proteins in intestinal organoids. On the other hand, *circHIPK3* silencing fails to alter IEC differentiation, because there are no significant differences in Paneth cells and enterocytes between *circHIPK3*-deficient organoids and control organoids. Studies conducted in an in vivo system further show that increasing the levels of *circHIPK3* by infecting mice with a recombinant *circHIPK3* lentiviral expression vector promotes the growth of the small intestinal mucosa, since it markedly increases the proliferating crypt cell population in mice. In contrast, overexpression of *circHIPK3* does not alter enterocyte migration along the crypt–villus axis as examined by BrdU pulse–chase assays. Together, these findings indicate that *circHIPK3* is crucial for the growth of the intestinal mucosa and wound healing after acute injury, although it plays a smaller role in the regulation of IEC differentiation.

### 3.2. Cdr1as

*Cdr1as* is a circularized antisense transcript derived from the *Cdr1* gene, and it is highly conserved across mammals [7,56]. Because *Cdr1as* is efficiently circularized during its biogenesis, *Cdr1as* is not detectable as a linear transcript in human and mouse cells. It has been shown that *Cdr1as* harbors >70 binding sites for miRNA-7 (miR-7) and acts as a sponge for miR-7 by reducing the number of freely available miR-7 molecules in human cells [7,8]. *CDR1as* also contains a full complementary binding site for miR-671, but the association of *CDR1as* with miR-671 is to slice *CDR1as* [57]. The basal levels of *CDR1as* are relatively low, but its expression levels increase dramatically in many tissues and organs responding to various pathological stresses such as inflammation, injury, and cancers [13,58]. Genetic ablation of the *Cdr1as* locus from the mouse genome decreases miR-7 levels in brain tissues and derepresses the production of miR-7-regulated proteins, thus affecting brain function [59]. Until recently, *Cdr1as* was found to play an important role in regulating intestinal epithelium homeostasis, specifically its implication in gut mucosal repair after acute injury [14].

Targeted deletion of the *Cdr1as* locus promotes renewal and defense of the intestinal epithelium in mice. Using a loss-of-function model for *Cdr1as* by ablating the mouse *Cdr1as* locus with CRISPR-Cas9, we have reported that *Cdr1as* knockout (*Cdr1as*^−/−^) mice exhibit a substantial increase in proliferating crypt cell population and longer villi and crypts in the small intestine [14]. Analysis of intestinal stem cell (ISC) activity further reveals that the small intestinal mucosa displays increased numbers of Olfm4- and Lgr5-positive cells in *Cdr1as*^−/−^ mice relative to littermates, suggesting that ISCs are activated in the *Cdr1as*-deficient intestinal epithelium. Decreasing the levels of *Cdr1as* also promotes the function of Paneth cells, Goblet cells, and Tuft cells in the intestinal epithelium, although it fails to alter enterocyte differentiation as examined by villin immunostaining analysis. *Cdr1as* deletion does not alter the abundances of mucosal tissue Pou2f3, Hes1, Atoh1, or Sox9 proteins; all are transcription factors and regulators necessary for differentiation of secretory lineage cells in the intestinal mucosa [1,2]. These results suggest that *Cdr1as* regulates the function of Paneth, Goblet, and Tuft cells by altering the overall renewal of the epithelium, rather than by specifically stimulating these transcriptional factors and regulators. Moreover, targeted deletion of the *Cdr1as* locus improves gut barrier function, since *Cdr1as*^−/−^ mice exhibit a lower basal level of gut permeability than that observed in control littermates, as measured by FITC-conjugated dextran assays [49].

Consistent with its effect on the epithelial renewal and gut barrier function, *Cdr1as*^−/−^ mice display increased levels of proliferation-associated proteins Pcna and cyclin-dependent kinase 6 (Cdk6), adherens junction protein E-cadherin, and tight junction proteins claudin-3 and claudin-1 in the small intestinal mucosa [14]. Since autophagy is essential for host defense of the intestinal epithelium against invasive pathogens and for gut barrier function [48,55], studies further examined the levels of autophagy-related proteins and demonstrated that loss of *Cdr1as* does not alter the expression levels of autophagy-related genes (ATGs), although it substantially reduces Beclin 1 levels in the intestinal mucosa.

In studies conducted in an ex vivo system, intestinal organoids derived from *Cdr1as*^−/−^ mice grow much faster than those generated from littermate mice. There is a substantial increase in the intestinal organoid surface area and BrdU incorporation in *Cdr1as*-deficient organoids compared with organoids from control littermates [14]. The activity of ISCs in the *Cdr1as*-deficient intestinal organoids also increases significantly, as examined by immunohistochemical detection of ISC markers Olfm4 and Lgr5. Consistently, intestinal organoids derived from *Cdr1as*^−/−^ mice exhibit increased Paneth cells and Tuft cells relative to those observed in the organoids from littermate mice. These results clearly show that the accelerated renewal of intestinal epithelial cells in *Cdr1as*^−/−^ mice results primarily from a cell-autonomous regulatory effect of *Cdr1as* but not from the effect of secreted factors in the gut. Together, the results from these in vivo and ex vivo experiments support the notion that *Cdr1as* is a negative regulator of the intestinal epithelial renewal and differentiation.

*Cdr1as* also functions as a repressor of intestinal mucosal repair after acute injury. Experiments using the mesenteric ischemia/reperfusion (I/R)-induced mucosal injury model reveal that both littermates and *Cdr1as*^−/−^ mice subjected to mesenteric I/R exhibit signs of markable mucosal injury and erosions in the small intestine, as evidenced by denuded villi with dilated capillaries, severe sloughed cells, and by frank hemorrhage microscopically. However, *Cdr1as* deletion decreases the degrees of I/R-induced mucosal injury in the small intestine, since the injury scores in *Cdr1as*^−/−^ mice are lower than those observed in littermates when measured immediately after mesenteric I/R [14]. Furthermore, *Cdr1as*-deficient intestinal epithelium exhibits an increase in mucosal repair after acute injury. Consistently, ablation of the *Cdr1as* locus also promotes the recovery of gut barrier function after I/R-induced stress. In the dextran sulfate sodium (DSS)-induced colitis model, *Cdr1as* deletion protects the colonic mucosa against DSS-induced injury, since there are only minor mucosal injuries/erosions observed in *Cdr1as*^−/−^ mice on day 7 after treatment with 3% DSS in drinking water. *Cdr1as* knockout also protects the colonic mucosa against DSS-induced apoptosis, as revealed by TUNEL staining. These findings strongly suggest that decreasing the levels of *Cdr1as* not only enhances injury-induced epithelial regeneration but also protects the mucosa against colitis.

### 3.3. circPABPN1

*CircPABPN1* is derived from the *PABPN1* pre-mRNA and is a target of the RBP HuR that plays an important role in maintaining the intestinal epithelium homeostasis [12,31]. Although HuR does not affect *circPABPN1* levels, *circPABPN1* remarkably inhibits HuR binding to *PABPN1* mRNA and represses PABPN1 translation in human cervical carcinoma HeLa cells [31]. We have demonstrated that *circPABPN1* is involved in intestinal epithelium-host defense and mucosal integrity by regulating autophagy via interaction with HuR [12]. Intestinal epithelial autophagy is crucial for mucosal defense against invasive pathogens, and its defects occur frequently in patients with various inflammatory mucosal disorders [60]. Autophagy facilitates the clearance of damaged organelles, regulates inflammatory responses, and provides nutrients during metabolic stress. More than 30 autophagy-related proteins are identified in mammals, and Atg16l1, a product of the *Atg16l1* gene, plays an important role in intestinal epithelium homeostasis [61]. ATG16L1 also inhibits necroptosis in the intestinal epithelium [62] and protects against TNF-induced apoptosis during chronic colitis in mice [63]. Mutations in autophagy genes, particularly *Atg16l1*, are associated with increased susceptibility to Crohn’s disease [64].

*CircPABPN1* prevents HuR binding to the *Atg16l1* mRNA and inhibits ATG16L1 production in the intestinal epithelium [12]. Consistent with the findings in HeLa cells, HuR directly interacts with *circPABPN1* in cultured human IECs. Ectopically expressed *circPABPN1* inhibits the expression of ATG16L1 without affecting the levels of *ATG16L1* mRNA. Studies examining the role of the *circPABPN1*-HuR complex in the regulation of ATG16L1 expression show that the ectopic rise in *circPABPN1* levels abolishes the binding of HuR to the 3′-untranslated region (3′-UTR) of *Atg16l1* mRNA. In accordance with these effects, ectopically expressed HuR partially but significantly rescues ATG16L1 expression in cells overexpressing *circPABPN1*. In contrast, HuR silencing and *circPABPN1* overexpression synergistically inhibit ATG16L1 expression, since the levels of ATG16L1 in cells co-transfected with *circPABPN1* expression vector and HuR silencer are lower than those observed in cells transfected with HuR silencer or *circPABPN1* expression vector alone. Interestingly, human mucosal tissues obtained from patients with ulcerative colitis exhibit a significant increase in the levels of *circPABPN1*, along with a decrease in the abundance of mucosal ATG16L1. Taken together, these findings indicate that *circPABPN1* and HuR regulate ATG16L1 expression antagonistically and that increased levels of tissue *circPABPN1* decrease ATG16L1 levels predominantly by suppressing HuR binding to the *ATG16L1* mRNA.

### 3.4. CircGMCL1 and CircHECTD1

*CircGMCL1* (hsa_circ_0055097) functions as a protective effector in the intestinal epithelium [32]. The levels of tissue *circGMCL1* decrease significantly in the colonic mucosa from patients with Crohn’s disease. Mechanistically, *circGMCL1* acts as a molecular sponge for miR-124-3p in the intestinal epithelium, thereby derepressing annexin A7 (ANXA7), which is a target of miR-124-3p. ANXA7 enhances autophagic flux in intestinal epithelial cells and promotes autophagy through the *circGMCL1*/miR-124-3p/ANXA7 axis. Ectopically expressed *circGMCL1* ameliorates injury severity and reduces pro-inflammatory cytokine production in the DSS-induced colitis model, and it also improves gut barrier function.

*CircHECTD1* is highly expressed in the intestinal epithelium, but its levels decrease in colonic tissues from patients with ulcerative colitis [33]. *CircHECTD1* sequesters miR-182-5p, which normally targets HuR. By reducing miR-182-5p availability, *circHECTD1* increases HuR expression by stabilizing its mRNA, leading to an increase in the levels of ATG5 and ATG9. Overexpression of *circHECTD1* in mice with DSS-induced colitis reduces colonic mucosal injuries and inflammation by promoting HuR-dependent autophagy.

### 3.5. CircPan3 and CircBtnl1

*CircPan3* is highly expressed in LGR5-positive ISCs located in the intestinal crypts and plays an important role in maintaining ISC proliferation [34]. Targeted deletion of *circPan3* in LGR5-positive ISCs impairs their self-renewal capacity and compromises intestinal epithelial regeneration after injury. Mechanistically, *circPan3* binds to the *Il13ra1* mRNA, increasing its stability and leading to enhanced IL-13Rα1 expression on the ISC surface. IL-13, produced by group 2 innate lymphoid cells, engages IL-13Rα1 and activates IL-13/IL-13R signaling. This initiates Foxp1 expression, which associates with β-catenin to facilitate nuclear translocation, thus activating the Wnt/β-catenin pathway essential for LGR5-positive ISC maintenance. Genetic ablation of *circPan3* in mice exacerbates intestinal mucosal inflammation and injury, demonstrating its protective function in the gut epithelium homeostasis [34].

*CircBtnl1* functions as a negative regulator of ISC replication in the intestinal mucosa, whereas loss of *circBtnl1* enhances ISC self-renewal capacity and promotes epithelial regeneration [35]. Mechanistically, *circBtnl1* and *Atf4* mRNA competitively bind the ATP-dependent RNA helicase Ddx3y, and this competitive binding impairs *Atf4* mRNA stability, inhibiting Atf4 protein expression. Atf4 is a transcription factor that activates the transcription of SOX9, which is essential for ISC maintenance and Paneth cell differentiation [36]. In *circBtnl1* knockout ISCs, loss of competition for Ddx3y binding promotes *Atf4* mRNA stability, increases Atf4 protein levels, and subsequently enhances SOX9 expression.

### 3.6. CircCDKN2B-AS1 and CircSOD2

*CircCDKN2B-AS1* is expressed mainly in colonic epithelial cells and has been shown to regulate tight junction protein expression [37]. Deletion of *circCDKN2B-AS1* enhances gut barrier function by altering the expression of tight junctions, including claudin-2. The levels of tissue *circCDKN2B-AS1* decrease remarkably in human intestinal mucosal tissues from patients with IBD.

*CircSOD2* is derived from back-splicing of exons 3 and 5 of the *superoxide dismutase 2* gene, and its levels increase dramatically in the intestinal mucosa from patients with ulcerative colitis [38]. Functional studies demonstrate that *circSOD2* overexpression disrupts gut barrier function, since it increases paracellular permeability and reduces tight junction proteins, including ZO-1, occludin, and claudin-1.

## 4. Mechanisms Underlying CircRNAs in the Intestinal Epithelial Homeostasis

An increasing body of evidence indicates that circRNAs regulate gene expression through diverse molecular mechanisms in maintaining the intestinal epithelium homeostasis [12,13,14,32,33,65]. Although many questions remain unclear and more experiments are needed, three principal mechanisms are proposed in general: (1) serving as competing endogenous RNAs (ceRNAs) or miRNA sponges; (2) interacting with RBP; and (3) modulating gene transcription.

### 4.1. CircRNAs’ Function as miRNA Sponges and Regulators

MiRNAs are small ncRNAs of ~22 nucleotides and have been shown to repress the expression patterns of various genes by directly interacting with the 3′-UTRs of target mRNAs [66]. High-throughput and functional studies have revealed that miRNAs are implicated in many aspects of different cellular processes and play an essential role in the pathogenesis of many human disorders, including tissue injury/repair, heart malfunctions, inflammation, and cancers [65,66,67,68,69,70,71,72]. Although miRNAs are powerful, with each molecule often able to regulate more than one target, and, vice versa, mRNAs are commonly targeted by several miRNAs. As such, miRNAs play an important role in the intestinal epithelium homeostasis and are involved in the regulation of the epithelial renewal and gut barrier function [67,68]. Global miRNA profiling studies show that expression levels of several miRNAs, including miRNA-29b, miR-195, miRNA-222, miRNA-322, miR-503, and miR-675, are dramatically altered in the intestinal epithelium after exposure to biological and pathological stresses such as food starvation, septic stress, and polyamine depletion [68,69,70]. The most extensively characterized functions of circRNAs are their ability to act as miRNA sponges through the competing ceRNA mechanism [8,71,72]. CircRNAs are particularly well-suited for this role due to their high stability, resistance to degradation, and enrichment with multiple miRNA response elements.

#### 4.1.1. CircHIPK3 Directly Binds to miR-29b and Inhibits miR-29b Activity

Since *circHIPK3* is predominantly distributed in the cytoplasm of IECs, we had tested the possibility that *circHIPK3* regulates the intestinal epithelium homeostasis by interacting with specific miRNAs [13]. Through searching for *circHIPK3*-interacting miRNAs using the TargetScan algorithm, miR-29b was found to be a potential target of *circHIPK3*, as there are several potential miR-29b binding sites on *circHIPK3*. Based on the fact that miR-29b-1-3p (miR-29b) is highly expressed in IECs and plays an important role in the control of intestinal epithelial renewal and gut barrier function [73,74], we examined the direct interaction between these two ncRNAs by using a biotinylated miR-29b assay [13]. As expected, the levels of *circHIPK3* in samples pulled down using biotin-labeled miR-29b are much higher than those in samples pulled down by using biotin-labeled scramble oligomer. On the other hand, biotin-labeled miR-29b does not pull down other circRNAs such as *Cdr1as* or *circPABPN1*. The miRNA-luciferase reporter assay using the vector expressing chimeric RNA that contains the luciferase coding region and a 3′-UTR bearing two perfect target sequences of miR-29b reveals that the luciferase activity decreases in *circHIPK3*-deficient cells when transfected with the miR-29b reporter vector, primarily resulting from an increasing availability of miR-29b by *circHIPK3* silencing. In contrast, luciferase activity increases in cells overexpressing *circHIPK3*, due to the decreased activity of miR-29b. These findings strongly suggest that *circHIPK3* sequesters and inhibits miR-29b activity in the intestinal epithelium.

Interaction between *circHIPK3* and miR-29b regulates epithelial repair after wounding [13]. Experiments examining the association of miR-29b with mRNAs encoding epithelial repair-associated proteins show that miR-29b directly binds to the mRNAs of *Rac1*, *Cdc42*, and *cyclin B1* but not mRNAs encoding RhoA, Cdk4, and p21. Increasing the levels of miR-29b inhibits the expression of Rac1, Cdc42, and cyclin B1, but this inhibition is prevented by overexpressing *circHIPK3*. Consistent with its stimulatory effect on the expression of Rac1, Cdc42, and cyclin B1, the elevation of cellular *circHIPK3* levels rescues the formation of membrane protrusions and restores the epithelial repair in cells overexpressing miR-29b. Interestingly, the levels of *circHIPK3* increase rapidly after wounding, which is associated with a decrease in the levels of miR-29b. These findings demonstrate that by modulating the expression of these specific target transcripts, at least in part through miR-29b, the elevation of cellular *circHIPK3* enhances cell migration and proliferation during epithelial repair after wounding, thereby contributing to the maintenance of the intestinal epithelial integrity under various stressful environments.

#### 4.1.2. Regulation of miR-195 by Cdr1as

*Cdr1as* affects intestinal epithelium homeostasis by modulating functions of several miRNAs, based on studies using microarray-based interrogation of global miRNA expression profiles [14]. A comparison of the miRNA expression profiles in *Cdr1as*^−/−^ mice relative to control littermates reveals that 46 miRNAs are differentially expressed in the small intestinal mucosa after *Cdrlas* deletion. In *Cdr1as*^−/−^ mice, 28 miRNAs decrease, including miR-351, miR-195-3p, miR-7, miR-195-5p, miR-489-3p, miR-1983, miR-5625, miR-7047, miR-21b, and miR-7069, while 18 miRNAs, including miR-412, miR-7039, miR-879, miR-6357, and miR-671, increase when compared with control littermate mice. Quantitative real-time polymerase chain reaction (Q-PCR) analysis confirms that loss of *Cdr1as* significantly lowers the levels of miR-195 and miR-7 in the small intestinal mucosa, but it increases the abundances of miR-671 and miR-412 without affecting the levels of miR-29b and miR-222. In cultured human IECs, inducing *Cdr1as* levels results in a remarkable increase in the levels of miR-195 that influences mucosal growth [75,76,77] and epithelial repair after acute injury [55]. Ectopic overexpression of the *Cdr1as* also inhibits intestinal epithelial repair after wounding, whereas neutralization of miR-195 by transfection with a miR-195 antagomir restores epithelial repair in *Cdr1as*-overexpressing cells. In an ex vivo model, *Cdr1as* overexpression also markedly inhibits the growth of intestinal organoids, and this inhibition is abolished by antagonizing miR-195. These findings indicate that *Cdr1as* decreases intestinal epithelial repair after wounding and inhibits mucosal renewal to a large extent by enhancing miR-195 function.

*Cdr1as* regulates miRNA biogenesis and stabilizes miR-195 precursor in the intestinal epithelium [14]. Studies examining changes in the levels of primary (pri-) and precursor (pre)-miR-195 transcripts in the small intestinal mucosa of *Cdr1as*^−/−^ and littermate mice reveal that loss of the *Cdr1as* locus in mice does not alter levels of tissue pri-miR-195, but it decreases pre-miR-195 abundance in the intestinal mucosa. In contrast, deletion of the *Cdr1as* locus fails to alter the levels of pre-miR-29b in the intestinal mucosa. These results indicate that *Cdr1as* regulates miR-195 expression through mechanisms other than transcription of pri-miR-195. Interestingly, *Cdr1as* is involved in the regulation of miRNA processing in the intestinal epithelium, since targeted deletion of *Cdr1as* in mice reduces the Dicer complex, markedly lowering the levels of Dicer and Trbp proteins in the intestinal mucosa of *Cdr1as*^−/−^ mice relative to control littermates. In addition, *Cdr1as*^−/−^ mice also show a selective decrease in LIN28 levels, but not in Ago2 levels, in the intestinal mucosa. On the other hand, *Cdrlas* deletion does not affect Drosha-mediated miRNA processing in the nucleus. Because Dicer function is essential for miRNA processing in the cytoplasm, a decrease in the levels of Dicer and Trbp proteins in the *Cdr1as*-deficient intestinal epithelium may contribute to the deregulation of miR-195 biogenesis.

Experiments examining the turnover of pre-miR-195 show that ectopically overexpressed *Cdr1as* increases the levels of miR-195 partially by altering pre-miR-195 stability [14]. This regulatory effect of *Cdr1as* on pre-miR-195 stability is specific, as increasing the levels of cellular *Cdr1as* does not alter the half-life of pre-miR-29b in IECs. Together, these results strongly suggest that *Cdr1as* upregulates miR-195 expression at least partially by stabilizing pre-miR-195 and enhancing the processing from pre-miR-195 to mature miR-195 in the intestinal epithelium.

### 4.2. Interaction Between CicrRNAs and RBPs

RBPs contain structural motifs such as RNA recognition motif and dsRNA binding domain, by which RBPs specifically bind to target mRNAs to alter their stability and translation negatively or positively [67,69,78]. Some RBPs have housekeeping functions, but many RBPs bind to specific subsets of mRNAs and control gene expression levels in response to various pathobiological stresses [79]. Hu/embryonic lethal and abnormal vision (ELAV) family of RBPs, including HuB, HuC, HuD, and HuR, stabilizes mRNAs and enhances translation of target mRNAs, whereas RBPs, such as AU-binding factor 1, CUG-binding protein 1, and tristetraprolin, increase the decay of target transcripts [80,81]. Significant changes in the binding affinity of RBPs for target mRNAs, their altered subcellular distribution, mutations in RBP binding regions, and deregulation of RBP production occur commonly in various pathological conditions and are involved in different aspects of many human disorders [82,83]. HuR is one of the best studied RBPs and plays a critical role in maintaining the intestinal epithelium homeostasis by actively participating in the regulation of various biological processes, including epithelium-host defense, gut barrier function, mucosal growth, and injury-induced epithelial regeneration [84,85,86].

CircRNAs interact extensively with RBPs and can serve as protein sponges that sequester RBPs as scaffolds facilitating protein–protein interactions, as decoys preventing RBPs from accessing target transcripts, and/or as recruiters bringing RBPs to particular cellular locations [24,87]. HuR exemplifies the importance of circRNA-RBP interactions in the intestinal epithelium. HuR is associated with multiple circRNAs, including *circPABPN1*, *circHECTD1*, and *Cdr1as* [12,14,33]. *CircPABPN1* directly interacts with HuR, alters HuR binding affinity for the *Atg16l1* mRNA via its 3′-UTR, and abolishes HuR-mediated ATG16L1 translation, thus influencing autophagy in the intestinal epithelium [12]. HuR also stabilizes the mRNAs encoding ATG5 and ATG9, but there are no studies available showing that *circPABPN1* affects HuR binding to the *Atg5* and *Atg9* mRNAs in the gut mucosa. In addition, *circHECTD1* regulates HuR protein levels by sponging miR-182-5p [33]. In mice with intestinal epithelial-specific deletion of HuR, the levels of tissue *Cdr1as* in the mucosa increase markedly, suggesting that HuR might function as a suppressor of *Cdr1as* expression [14].

*CircBtnl1* exhibits the protein scaffold function and can bind to the RBP Ddx3y, and this interaction between *circBtnl1* and Ddx3y decreases Ddx3y binding to the *Atf4* mRNA [35]. Decrease in the levels of the Ddx3y/*Atf4* mRNA complex by *circBtnl1* overexpression destabilizes the *Atf4* transcript and decreases Atf4 protein levels, subsequently inhibiting the expression of SOX9 and IEC differentiation. Quaking (QKI) represents an RBP that regulates circRNA biogenesis, and its levels are also regulated by circRNAs [24,88]. QKI binds to QKI response elements in flanking introns and promotes circRNA formation. In the gut mucosa, QKI-regulated circRNA production plays an important role in the control of IEC differentiation [89]. Interestingly, individual circRNA often regulates target genes via multiple mechanisms simultaneously [24].

### 4.3. Transcriptional Regulation

It has been reported that nuclear-localized circRNAs, particular circular intronic RNAs (ciRNAs) and exon–intron circRNAs, are implicated in modulating the transcription of their parent genes by interacting with RNA polymerase II or transcriptional machinery [17,18]. CircRNAs accumulate at their transcription sites and enhance RNA polymerase II activity, thus altering their host gene transcription. On the other hand, several circRNAs are shown to be translated to small peptides and proteins through internal ribosome entry sites or m6A-mediated mechanisms [88,89]. N6-methyladenosine (m6A) modifications on circRNAs can recruit translation initiation factors, enabling cap-independent translation [28]. However, it remains unclear whether any circRNA can produce active and functional small peptides and proteins in regulating the gut epithelium homeostasis.

**Figure 2:**
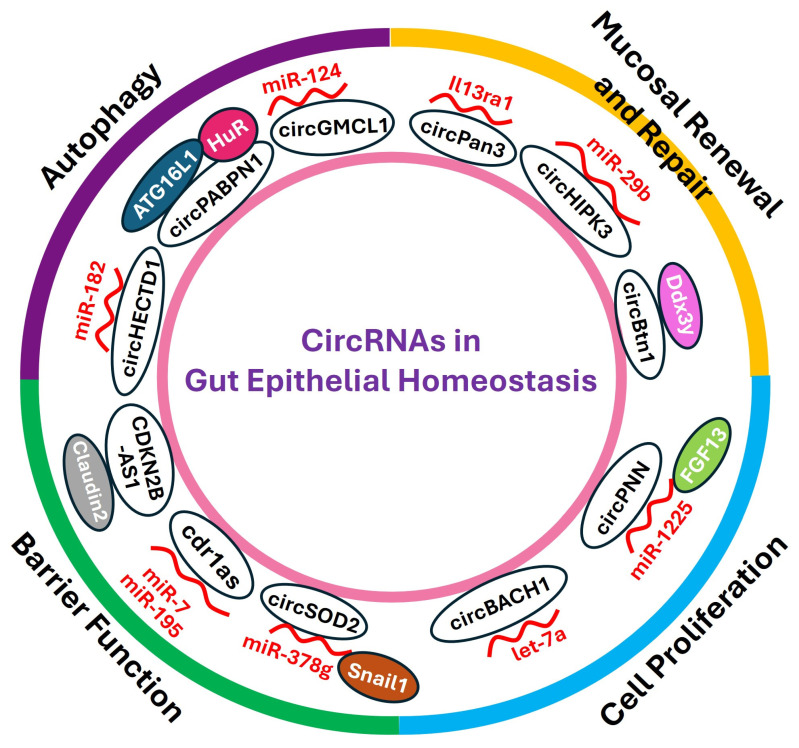
CircRNAs in maintaining the intestinal epithelium homeostasis. CircRNAs influence the integrity of the intestinal epithelium by altering constant mucosal renewal, epithelial repair after acute injury, autophagy, ISC proliferation, and gut barrier function via interactions with miRNAs and RBPs. Increased levels of a group of circRNAs such as *circHIPK3* and *circGMCL1* protect the epithelium against stress and injury, whereas others such as *Cdr1as* and *circPABPN1* induce intestinal mucosal disruption.

Taken together, the results obtained from these in vitro, ex vivo, and in vivo experiments strongly suggest that circRNAs modulate gene regulatory programs governing the intestinal epithelium homeostasis by altering mucosal growth and repair, autophagy, gut barrier function, and ISC proliferation via interactions with miRNAs and RBPs (Figure 2). Based on this model, *circHIPK3*, *Cdr1as*, *circGMCL1*, *circHECTD1*, *circPan*, *circBACH1* [39], *circPNN* [40], *CDKN2B-AS1*, and *CircSOD2* regulate mucosal growth, injury-induced epithelial regeneration, and gut barrier function by acting as sponges for target miRNAs or/and altering miRNA biogenesis in the intestinal epithelium, while *circPABPN1* and *circBtnl1* affect autophagy and proliferation by interacting with RBPs. These exciting findings demonstrating the association of circRNAs with miRNAs and RBPs advance our understanding of circRNAs and their biological functions in the intestinal epithelium homeostasis and in response to various pathological stresses.

Although miRNA sponging, RBP interactions, and transcriptional regulation have been discussed separately, these mechanisms are not mutually exclusive and function as interconnected regulatory layers in intestinal epithelial cells. Among them, miRNA sponging is the most extensively documented, reflecting the predominantly cytoplasmic localization of circRNAs in epithelial tissues [11]. Notably, certain circRNAs simultaneously engage miRNA sponging and RBP interactions, functioning as multivalent scaffolds that integrate multiple regulatory signals, collectively underscoring the functional versatility of circRNAs in maintaining intestinal epithelial homeostasis [16].

## 5. Implication of CircRNAs in Gut Mucosal Disorders

Dysregulation of circRNA expression has been implicated in various gut mucosal disorders, reflecting its importance in maintaining the intestinal epithelium homeostasis and responding to pathologies. This section highlights and is highly focused on the roles of circRNAs in gut mucosal inflammatory injury diseases, surgical disorders, and gastrointestinal (GI) malignancies.

### 5.1. IBD

IBD encompasses Crohn’s disease and ulcerative colitis, both characterized by chronic relapsing inflammation [90,91]. CircRNA dysregulation has been well documented in IBD and contributes to IBD pathogenesis through the regulation of gut barrier function, injury/repair, autophagy, immune activity, and inflammatory responses [11,92]. To explore the implications of altered circRNAs clinically, small intestine and colonic mucosal tissues from patients with Crohn’s disease and ulcerative colitis were collected, and tissue samples from patients without gut mucosal injury/erosions, inflammation, or disrupted barrier served as controls [13]. Interestingly, intestinal mucosal tissues from patients with active Crohn’s disease and ulcerative colitis exhibit decreased levels of *circHIPK3*, as measured by Q-PCR and droplet digital Q-PCR analyses. RNA-FISH detection in the mucosa of the intestine reveals that *circHIPK3* is predominantly distributed in the cytoplasm of epithelial cells, and there are no significant differences in the *circHIPK3* expression levels between villi and crypts in control individuals. However, the intestinal mucosal tissues obtained from all patients with Crohn’s disease and ulcerative colitis exhibit a remarkable decrease in the levels of *circHIPK3*-FITC intensity. The decreased levels of the mucosal *circHIPK3* in patients with Crohn’s disease and ulcerative colitis are associated with severe mucosal injury/erosions, inflammation, delayed healing, and gut barrier dysfunction.

The levels of mucosal *circGMCL1* also decrease significantly in the colon of patients with Crohn’s disease, and these reduced levels of *circGMCL1* are correlated with inflammatory indices [32]. Therapeutic potential has been demonstrated using PLGA-microsphere delivery in IL-10 knockout mice, showing reduced colitis severity and improved gut barrier function by increasing *circGMCL1*. Decreased levels of *circHECTD1* are also observed in the colonic mucosa of patients with ulcerative colitis, whereas ectopically overexpressed *circHECTD1* reduces mucosal injuries in the colon by promoting HuR-dependent autophagy in mice [33]. In contrast, the levels of *Cdr1as* increase in the intestinal mucosal tissues in patients with Crohn’s disease and ulcerative colitis [14].

Deregulation of circRNAs is involved in gut barrier dysfunction in patients with IBD [37,92]. *CircSMAD4* sequesters miR-135a-5p, leading to JAK2 overexpression and degradation of tight junction proteins in patients with Crohn’s disease [11,41], while increased circ-SOD2 in patients with ulcerative colitis damages the intestinal epithelial barrier via the miR-378g/Snail1 pathway [38]. In addition, elevated *circRNA_103765* acts as a proinflammatory factor via sponging miR-30 family in patients with Crohn’s disease [42]. The structural stability, tissue-specific expression, and detectability of circRNAs via Q-PCR analysis make circRNAs ideal and potential candidates for biomarkers for patients with IBD [92,93,94]. Furthermore, since there are significant differences in the levels of circRNAs in serum between Crohn’s disease and ulcerative colitis, circRNAs can also serve as diagnostic markers and monitor for treatment response in patients with IBD [95].

### 5.2. I/R-Induced Mucosal Injury and Sepsis

Intestinal I/R-induced injury occurs in various clinical settings, including mesenteric arterial occlusion, small bowel transplantation, and hemorrhagic shock [96,97]. During intestinal I/R, oxidative stress and inflammation trigger multiple cell deaths, while circRNAs have emerged as important regulators and potential biomarkers for I/R-induced mucosal injury [98]. The levels of *circEZH2_005* decrease significantly in intestinal tissue exosomes isolated from intestinal I/R mice and in exosomes from the serum of patients who underwent cardiac surgery who are associated with postoperative I/R-induced injury in the intestine, suggesting a significant diagnostic value of exosomal *circEZH2_005* in patients with critical illnesses [43]. Mechanistically, *circEZH2_005* expressed in intestinal crypt cells promotes proliferation of LGR5-positive stem cells by direct interaction with hnRNPA1 and Gprc5a, thereby alleviating I/R-induced damage [93].

In patients with polymicrobial sepsis-induced intestinal epithelial injury, the levels of tissue *circHIPK3* decrease remarkably in the intestinal mucosa [13]. *CircHIPK3* acts as a stimulator for gut mucosal healing since the downregulation of *circHIPK3* inhibits cell proliferation and delays epithelial repair after wounding. Therefore, decreased levels of tissue *circHIPK3* may contribute to the pathogenesis of intestinal mucosal injury and delayed healing in patients with sepsis. Moreover, *circFLNA* is also involved in sepsis-induced gut mucosal injury, as evidenced by a significant increase in the levels of tissue *circFLNA* in the intestinal mucosa after exposure to sepsis [44]. *circFLNA* increases intestinal mucosal injury in sepsis through the Fas-mediated apoptosis pathway by sponging miR-766-3p [44].

### 5.3. GI Cancers

GI cancers represent a major cause of cancer-related mortality worldwide [43]. CircRNAs have been extensively studied in malignancies, demonstrating crucial roles in tumor development, progression, metastasis, and chemoresistance in the GI mucosa [99,100]. High-throughput sequencing studies have revealed extensive circRNA dysregulation in GI cancers [45,101]. In colorectal cancer, approximately 21,458 circRNAs are identified, with 448 differentially expressed compared to normal tissues. These circRNAs are widely involved in the regulation of cell communication, autophagosome formation, proliferation, migration, cancer invasions, and other cancer-relevant signaling pathways [45,99,100,101].

*CircCTNNA1* is upregulated in gastric cancer, which is associated with advanced tumor-node-metastasis stage and poor prognosis [45,46]. *CircCTNNA1* promotes cell proliferation, migration, and cancer invasion by sponging miR-363-3p. In contrast, *hsa_circ_0006633* is downregulated in GI cancer tissues and serves as a clinically useful diagnostic marker with high sensitivity and specificity [47]. *CircHIPK3* is also implicated in GI cancers and hepatocellular carcinoma by promoting tumorigenesis by regulating the miR-637/AKT1 pathway and by sponging miR-124 [30]. While circRNAs play indispensable roles in maintaining mucosal integrity, their expression profile undergoes significant reprogramming during the onset and progression of colorectal cancer (CRC). Emerging evidence highlights a pervasive downregulation of the global circRNA population in the early stages of colorectal tumorigenesis [102].

This depletion is not merely a byproduct of increased cell proliferation but is often driven by specific oncogenic signaling. For instance, KRAS mutations, a hallmark of CRC progression, have been shown to actively suppress circRNA biogenesis, leading to a reduction in transcripts that normally serve as tumor-suppressive sponges [103]. Interestingly, these altered circRNAs can also be sequestered into exosomes, suggesting that their depletion within the cell not only facilitates oncogenic signaling but also serves as a potential non-invasive biomarker for CRC detection [104]. The functional impact of circRNAs is inherently cell-type-specific. Recent single-cell transcriptomic analyses reveal that key circular transcripts and their host genes exhibit distinct expression patterns across the crypt–villus axis, with specific enrichment in Lgr5+ stem cells or specialized secretory lineages [104]. This granular distribution suggests that lineage-specific dysregulation may be a primary driver of mucosal barrier failure. CircRNAs can be easily detected in blood, serum, plasma, and tumor tissues with high sensitivity and specificity. Several circRNAs have been validated as diagnostic markers for early cancer detection, prognostic indicators, and predictive markers for treatment response. CircRNAs also represent potential therapeutic targets in GI cancers [105,106]. Various strategies, including antisense oligonucleotides targeting back-splice junctions, small molecule inhibitors against circRNA biogenesis, delivery of synthetic circRNAs, PLGA-microsphere delivery systems, and viral vector approaches, have shown promise and significant progress in several preclinical studies. However, there are many challenges in circRNA-guided therapeutics in cancers, which include functional characterization of differentially expressed circRNAs, clarification of cell-type-specific expression using single-cell approaches, optimization of delivery methods for tissue-specific targeting, and validation in large-scale clinical trials. With advancing knowledge of circRNA biology, it is likely that circRNA-focused strategies will play an important role in precision medicine for GI cancers and other mucosal disorders.

## 6. Summary and Conclusions

CircRNAs have emerged as critical regulators of intestinal epithelium homeostasis and diseases over the past decade. The unique structural features of circRNAs and their covalently closed loop lacking free ends confer extraordinary stability of circRNAs relative to linear RNAs. This long stability of circRNAs, together with their tissue-specific expression patterns in various stressful environments, points out that circRNAs are crucial regulators of physiology and pathology in the intestinal epithelium. The biogenesis of circRNAs in the gut mucosa is tightly regulated by multiple factors such as intron-pairing, RBP-mediated circularization, and lariat-driven processes. Intestinal mucosa-enriched circRNAs, including *circHIPK3*, *Cdr1as*, and *circPABPN1*, play an essential role in maintaining the intestinal epithelium homeostasis, whereas the deregulation of circRNA expression and function contributes to mucosal injury, delayed wound healing, gut barrier dysfunction, inflammation, and cancers. CircRNAs modulate the intestinal epithelium homeostasis and pathologies through multiple and distinct mechanisms, including sponging for miRNAs, interactions with RBPs, and regulation of gene transcription. Recent studies have identified numerous circular RNAs (circRNAs), including *circHIPK3* and *Cdr1as*, as regulators of intestinal epithelial biology; however, these findings should be interpreted with caution. Many studies rely on in vitro systems or limited in vivo models, often restricted to a single genetic background, which may affect the generalizability of results [16,22]. In addition, the role of the gut microbiota in intestinal homeostasis remains insufficiently explored in circRNA research [52]. Technical and conceptual challenges further limit the field, including concerns about reproducibility due to non-standardized analytical pipelines, as well as difficulties in circRNA detection and quantification related to low abundance and potential artifacts [107,108,109]. Moreover, current experimental models do not fully recapitulate human intestinal physiology, highlighting the need for more robust and translationally relevant approaches.

In this regard, there are still gaps in our knowledge and understanding of the biological functions of circRNAs in maintaining intestinal epithelium homeostasis and their clinical application. First, a better understanding of circRNA biogenesis, regulation, modifications, and cellular effects in response to different stresses is necessary and badly needed. Second, the functions of circRNAs expressed differentially in the intestinal mucosa in various pathophysiological conditions require systematic validation. Cell-type-specific expression of circRNAs and their pathobiological functions should be clarified and fully investigated through multiple state-of-the-art technologies, particularly in patients with critical illnesses. Third, we should define the mechanisms underlying the control of the IEC proliferation and cell-to-cell interaction by circRNAs, specifically their roles in the regulation of mRNA stability and translation during normal as well as pathological conditions. Fourth, the implication of small peptides and proteins translated potentially from circRNAs in the intestinal epithelium homeostasis and diseases requires rigorous elucidation in both in vitro and in vivo systems. Finally, while no specific diagnostic or therapeutic targets of circRNAs are clear in critically ill patients at this time, we should search for molecular signatures that help clinical application using circRNAs as biomarkers for acute disrupted integrity of the intestinal epithelium and systemic gut barrier dysfunction in clinical settings and as potential therapeutic targets for developing novel therapeutics to protect the gut epithelium against injury and inflammation in critically ill patients.

## Figures and Tables

**Table 1:** CircRNAs in intestinal mucosal homeostasis and pathologies. ↑ indicates the upregulation and ↓ indicates the downregulation.

Name	Functions	Targets	References
*circHIPK3*	↑ Intestinal epithelial repair after injury	Sponge of miR-29b	[13,29]
	↑ Mucosal renewal	cyclin B1	
	↑ Cell migration	Cdc42, Rac1, RhoA	
	↑ Tumorigenesis	miR-637/AKT1 pathway	[30]
	↑ Cancer growth	Sponge of miR-124	
*Cdr1as*	↓ Cell proliferation	Stabilizing miR-7, miR-195 E-cadherin, claudin-1 and claudin-3 CDK4 DICER, TRBP, LIN28	[14]
	↓ Function of ISC, Paneth cells, and Tuft cells		
	↓ Gut barrier function		
	↓ Injury-induced epithelial regeneration		
	↑ miRNA processing		
*circPABPN1*	↓ Autophagy	ATG16L1	[12,31]
	↓ Epithelium-host defense and mucosal integrity	HuR	
*circGMCL1*	↑ Autophagy	Sponge of miR-124	[32]
	↓ Pro-inflammatory cytokine production	ANXA7	
*circHECTD1*	↑ Autophagy	Sponge of miR-182	[33]
	↓ Mucosal injury and inflammation	HuR, ATG5, and ATG9	
*circPan3*	↓ ISC proliferation	IL13Ra1, Foxp1	[34]
	↓ Intestinal inflammation and injury		
*circBtnl1*	↓ ISC replication	Ddx3y	[35,36]
	↓ Epithelial regeneration and differentiation	ATF4, SOX9	
*circCDKN2B-AS1*	↓ Gut barrier function	Claudin-2	[37]
*circSOD2*	↓ Gut barrier function	miR-378g/Snail pathway	[38]
*circBACH1*	↑ Colorectal cancer cell proliferation	Let-7a-5p	[39]
*circPNN*	↑ Colorectal cancer growth	FGF13/miR-1225-5p pathway	[40]
*circSMAD4*	↓ Gut barrier function	Sponge of miR-135a-5p	[11,41]
*circRNA_103765*	↑ Pro-inflammatory factor	Sponge of miR-30 family	[42]
*circEZH2_005*	↑ Proliferation	hnRNPA1, Gprc4a	[43]
*circFLNA*	↑ Intestinal mucosal injury	Sponge of miR-766-3p	[44]
*circCTNNA1*	↑ Cancer cell proliferation, migration, and invasion	Sponge of miR-363-3p	[45,46]
*hsa_circ_0006633*	↓ GI cancer growth	Biomarker	[47]

## Data Availability

No new data were created or analyzed in this study. Data sharing is not applicable to this article.

## Abbreviations

IECs	intestinal epithelial cells;
RBPs	RNA-binding proteins;
IBD	inflammatory bowel diseases;
ANXA7	annexin A7;
ceRNAs	competing endogenous RNAs;
CLP	cecal ligation and puncture;
miRNA	microRNA;
UTRs	untranslated regions;
circRNAs	circular RNAs;
FITC	fluorescein isothiocyanate;
GI	gastrointestinal;
ISCs	intestinal stem cells;
*Cdr1as* ^−/−^	*Cdr1as* knockout;
DSS	dextran sulfate sodium;
I/R	ischemia/reperfusion.

## References

[1] Barker N., van de Wetering M., Clevers H. (2008). The intestinal stem cell. Genes Dev..

[2] van der Flier L.G., Clevers H. (2009). Stem cells, self-renewal, and differentiation in the intestinal epithelium. Annu. Rev. Physiol..

[3] Diener T.O. (1971). Potato spindle tuber “virus”. IV. A replicating, low molecular weight RNA. Virology.

[4] Sanger H.L., Klotz G., Riesner D., Gross H.J., Kleinschmidt A.K. (1976). Viroids are single-stranded covalently closed circular RNA molecules existing as highly base-paired rod-like structures. Proc. Natl. Acad. Sci. USA.

[5] Salzman J., Gawad C., Wang P.L., Lacayo N., Brown P.O. (2012). Circular RNAs are the predominant transcript isoform from hundreds of human genes in diverse cell types. PLoS ONE.

[6] Jeck W.R., Sorrentino J.A., Wang K., Slevin M.K., Burd C.E., Liu J., Marzluff W.F., Sharpless N.E. (2013). Circular RNAs are abundant, conserved, and associated with ALU repeats. RNA.

[7] Memczak S., Jens M., Elefsinioti A., Torti F., Krueger J., Rybak A., Maier L., Mackowiak S.D., Gregersen L.H., Munschauer M. (2013). Circular RNAs are a large class of animal RNAs with regulatory potency. Nature.

[8] Hansen T.B., Jensen T.I., Clausen B.H., Bramsen J.B., Finsen B., Damgaard C.K., Kjems J. (2013). Natural RNA circles function as efficient microRNA sponges. Nature.

[9] Salzman J., Chen R.E., Olsen M.N., Wang P.L., Brown P.O. (2013). Cell-type specific features of circular RNA expression. PLoS Genet..

[10] Rybak-Wolf A., Stottmeister C., Glazar P., Jens M., Pino N., Giusti S., Hanan M., Behm M., Bartok O., Ashwal-Fluss R. (2015). Circular RNAs in the mammalian brain are highly abundant, conserved, and dynamically expressed. Mol. Cell.

[11] Yang L., Li H., Tang M., He L., Yang L. (2025). Circular RNAs in inflammatory bowel disease: A review of mechanisms, biomarkers and therapeutic potential. Front. Immunol..

[12] Li X.X., Xiao L., Chung H.K., Ma X.X., Liu X., Song J.L., Jin C.Z., Rao J.N., Gorospe M., Wang J.Y. (2020). Interaction between HuR and circPABPN1 modulates autophagy in the intestinal epithelium by altering ATG16L1 translation. Mol. Cell. Biol..

[13] Xiao L., Ma X.X., Luo J., Chung H.K., Kwon M.S., Yu T.X., Rao J.N., Kozar R., Gorospe M., Wang J.Y. (2021). Circular RNA circHIPK3 promotes homeostasis of the intestinal epithelium by reducing microRNA 29b function. Gastroenterology.

[14] Chung H.K., Xiao L., Han N., Chen J., Yao V., Cairns C.M., Raufman B., Rao J.N., Turner D.J., Kozar R. (2024). Circular RNA Cdr1as inhibits proliferation and delays injury-induced regeneration of the intestinal epithelium. JCI Insight.

[15] Chen L.L. (2020). The expanding regulatory mechanisms and cellular functions of circular RNAs. Nat. Rev. Mol. Cell Biol..

[16] Kristensen L.S., Andersen M.S., Stagsted L.V.W., Ebbesen K.K., Hansen T.B., Kjems J. (2019). The biogenesis, biology and characterization of circular RNAs. Nat. Rev. Genet..

[17] Zhang Y., Zhang X.O., Chen T., Xiang J.F., Yin Q.F., Xing Y.H., Zhu S., Yang L., Chen L.L. (2013). Circular intronic long noncoding RNAs. Mol. Cell.

[18] Li Z., Huang C., Bao C., Chen L., Lin M., Wang X., Zhong G., Yu B., Hu W., Dai L. (2015). Exon-intron circular RNAs regulate transcription in the nucleus. Nat. Struct. Mol. Biol..

[19] Jeck W.R., Sharpless N.E. (2014). Detecting and characterizing circular RNAs. Nat. Biotechnol..

[20] Wang Y., Wang Z. (2015). Efficient backsplicing produces translatable circular mRNAs. RNA.

[21] Ashwal-Fluss R., Meyer M., Pamudurti N.R., Ivanov A., Bartok O., Hanan M., Evantal N., Memczak S., Rajewsky N., Kadener S. (2014). circRNA biogenesis competes with pre-mRNA splicing. Mol. Cell.

[22] Zhang X.O., Wang H.B., Zhang Y., Lu X., Chen L.L., Yang L. (2014). Complementary sequence-mediated exon circularization. Cell.

[23] Liang D., Wilusz J.E. (2014). Short intronic repeat sequences facilitate circular RNA production. Genes Dev..

[24] Conn S.J., Pillman K.A., Toubia J., Conn V.M., Salmanidis M., Phillips C.A., Roslan S., Schreiber A.W., Gregory P.A., Goodall G.J. (2015). The RNA binding protein quaking regulates formation of circRNAs. Cell.

[25] Enuka Y., Lauriola M., Feldman M.E., Sas-Chen A., Ulitsky I., Yarden Y. (2016). Circular RNAs are long-lived and display only minimal early alterations in response to a growth factor. Nucleic Acids Res..

[26] Szabo L., Salzman J. (2016). Detecting circular RNAs: Bioinformatic and experimental challenges. Nat. Rev. Genet..

[27] Liu C.X., Li X., Nan F., Jiang S., Gao X., Guo S.K., Xue W., Cui Y., Dong K., Ding H. (2019). Structure and degradation of circular RNAs regulate PKR activation in innate immunity. Cell.

[28] Zhou C., Molinie B., Daneshvar K., Pondick J.V., Wang J., Van Wittenberghe N., Xing Y., Giallourakis C.C., Mullen A.C. (2017). Genome-wide maps of m6A circRNAs identify widespread and cell-type-specific methylation patterns that are distinct from mRNAs. Cell Rep..

[29] Zheng Q., Bao C., Guo W., Li S., Chen J., Chen B., Luo Y., Lyu D., Li Y., Shi G. (2016). Circular RNA profiling reveals an abundant circHIPK3 that regulates cell growth by sponging multiple miRNAs. Nat. Commun..

[30] Yang D., Hu Z., Zhang Y., Zhang X., Xu J., Fu H., Zhu Z., Feng D., Cai Q. (2021). CircHIPK3 promotes the tumorigenesis and development of gastric cancer through miR-637/AKT1 pathway. Front. Oncol..

[31] Abdelmohsen K., Panda A.C., Munk R., Grammatikakis I., Dudekula D.B., De S., Kim J., Noh J.H., Kim K.M., Martindale J.L. (2017). Identification of HuR target circular RNAs uncovers suppression of PABPN1 translation by CircPABPN1. RNA Biol..

[32] Zhao J., Sun Y., Yang H., Qian J., Zhou Y., Gong Y., Dai Y., Jiao Y., Zhu W., Wang H. (2022). Correction: PLGA-microspheres-carried circGMCL1 protects against Crohn’s colitis through alleviating NLRP3 inflammasome-induced pyroptosis by promoting autophagy. Cell Death Dis..

[33] Xu Y., Tian Y., Li F., Wang Y., Yang J., Gong H., Wan X., Ouyang M. (2022). Circular RNA HECTD1 mitigates ulcerative colitis by promoting enterocyte autophagy via miR-182-5p/HuR axis. Inflamm. Bowel Dis..

[34] Zhu P., Zhu X., Wu J., He L., Lu T., Wang Y., Liu B., Ye B., Sun L., Fan D. (2019). IL-13 secreted by ILC2s promotes the self-renewal of intestinal stem cells through circular RNA circPan3. Nat. Immunol..

[35] Guo H., Zhang J., Jiang Z., Zhu X., Yang J., Mu R., Du Y., Tian Y., Zhu P., Fan Z. (2023). Noncoding RNA circBtnl1 suppresses self-renewal of intestinal stem cells via disruption of Atf4 mRNA stability. EMBO J..

[36] Cui C., Wang F., Zheng Y., Wei H., Peng J. (2023). From birth to death: The hardworking life of Paneth cell in the small intestine. Front. Immunol..

[37] Rankin C.R., Lokhandwala Z.A., Huang R., Pekow J., Pothoulakis C., Padua D. (2019). Linear and circular CDKN2B-AS1 expression is associated with Inflammatory Bowel Disease and participates in intestinal barrier formation. Life Sci..

[38] Ye G., Zhang J., Peng J., Zhou Z., Wang W., Yao S. (2024). CircSOD2: Disruption of intestinal mucosal barrier function in ulcerative colitis by regulating the miR-378g/Snail1 axis. J. Gastroenterol. Hepatol..

[39] Li J., Tang Q., Dong W., Wang Y. (2020). CircBACH1/let-7a-5p axis enhances the proliferation and metastasis of colorectal cancer by upregulating CREB5 expression. J. Gastrointest. Oncol..

[40] Liao X., Li T., Yang L., Li H., Li W., Liu Y., Xie Z. (2024). Tumor-derived exosomal circular RNA Pinin induces FGF13 expression to promote colorectal cancer progression through miR-1225-5p. Gut Liver.

[41] Wang T., Chen N., Ren W., Liu F., Gao F., Ye L., Han Y., Zhang Y., Liu Y. (2019). Integrated analysis of circRNAs and mRNAs expression profile revealed the involvement of hsa_circ_0007919 in the pathogenesis of ulcerative colitis. J. Gastroenterol..

[42] Ye Y., Zhang L., Hu T., Yin J., Xu L., Pang Z., Chen W. (2021). CircRNA_103765 acts as a proinflammatory factor via sponging miR-30 family in Crohn’s disease. Sci. Rep..

[43] Zhang W., Zhou B., Yang X., Zhao J., Hu J., Ding Y., Zhan S., Yang Y., Chen J., Zhang F. (2023). Exosomal circEZH2_005, an intestinal injury biomarker, alleviates intestinal ischemia/reperfusion injury by mediating Gprc5a signaling. Nat. Commun..

[44] Ye L., Shi Y., Zhang H., Chen C., Niu J., Yang J., Li Z., Shao H., Qin B. (2023). circFLNA promotes intestinal injury during abdominal sepsis through Fas-mediated apoptosis pathway by sponging miR-766-3p. Inflamm. Res..

[45] Zhang Y., Zheng S., Liao N., Huang H., Chen W., Wu Z., Wu D. (2021). CircCTNNA1 acts as a ceRNA for miR-363-3p to facilitate the progression of colorectal cancer by promoting CXCL5 expression. J. Biol. Res..

[46] Fang L., Du W.W., Lyu J., Dong J., Zhang C., Yang W., He A., Kwok Y.S.S., Ma J., Wu N. (2018). Enhanced breast cancer progression by mutant p53 is inhibited by the circular RNA circ-Ccnb1. Cell Death Differ..

[47] Lu R., Shao Y., Ye G., Xiao B., Guo J. (2017). Low expression of hsa_circ_0006633 in human gastric cancer and its clinical significances. Tumour Biol..

[48] Yu T.X., Chung H.K., Xiao L., Piao J.J., Lan S., Jaladanki S.K., Turner D.J., Raufman J.P., Gorospe M., Wang J.Y. (2020). Long noncoding RNA H19 impairs the intestinal barrier by suppressing autophagy and lowering paneth and goblet cell function. Cell. Mol. Gastroenterol. Hepatol..

[49] Yu T.X., Wang P.Y., Rao J.N., Zou T., Liu L., Xiao L., Gorospe M., Wang J.Y. (2011). Chk2-dependent HuR phosphorylation regulates occludin mRNA translation and epithelial barrier function. Nucleic Acids Res..

[50] Li G.X., Wang X.M., Jiang T., Gong J.F., Niu L.Y., Li N. (2014). Berberine prevents damage to the intestinal mucosal barrier during early phase of sepsis in rat through mechanisms independent of the NOD-like receptors signaling pathway. Eur. J. Pharmacol..

[51] Chen S., He Y., Hu Z., Lu S., Yin X., Ma X., Lv C., Jin G. (2017). Heparanase mediates intestinal inflammation and injury in a mouse model of sepsis. J. Histochem. Cytochem..

[52] Li X., Yang L., Chen L.L. (2018). The biogenesis, functions, and challenges of circular RNAs. Mol. Cell.

[53] Li Y., Zheng F., Xiao X., Xie F., Tao D., Huang C., Liu D., Wang M., Wang L., Zeng F. (2017). CircHIPK3 sponges miR-558 to suppress heparanase expression in bladder cancer cells. EMBO Rep..

[54] Liu L., Zhuang R., Xiao L., Chung H.K., Luo J., Turner D.J., Rao J.N., Gorospe M., Wang J.Y. (2017). HuR enhances early restitution of the intestinal epithelium by increasing Cdc42 translation. Mol. Cell. Biol..

[55] Zhuang R., Rao J.N., Zou T., Liu L., Xiao L., Cao S., Hansraj N.Z., Gorospe M., Wang J.Y. (2013). miR-195 competes with HuR to modulate stim1 mRNA stability and regulate cell migration. Nucleic Acids Res..

[56] de Chevigny A., Core N., Follert P., Gaudin M., Barbry P., Beclin C., Cremer H. (2012). miR-7a regulation of Pax6 controls spatial origin of forebrain dopaminergic neurons. Nat. Neurosci..

[57] Hansen T.B., Wiklund E.D., Bramsen J.B., Villadsen S.B., Statham A.L., Clark S.J., Kjems J. (2011). miRNA-dependent gene silencing involving Ago2-mediated cleavage of a circular antisense RNA. EMBO J..

[58] Kristensen L.S., Ebbesen K.K., Sokol M., Jakobsen T., Korsgaard U., Eriksen A.C., Hansen T.B., Kjems J., Hager H. (2020). Spatial expression analyses of the putative oncogene ciRS-7 in cancer reshape the microRNA sponge theory. Nat. Commun..

[59] Piwecka M., Glazar P., Hernandez-Miranda L.R., Memczak S., Wolf S.A., Rybak-Wolf A., Filipchyk A., Klironomos F., Cerda Jara C.A., Fenske P. (2017). Loss of a mammalian circular RNA locus causes miRNA deregulation and affects brain function. Science.

[60] Hampe J., Franke A., Rosenstiel P., Till A., Teuber M., Huse K., Albrecht M., Mayr G., De La Vega F.M., Briggs J. (2007). A genome-wide association scan of nonsynonymous SNPs identifies a susceptibility variant for Crohn disease in ATG16L1. Nat. Genet..

[61] Mizushima N., Komatsu M. (2011). Autophagy: Renovation of cells and tissues. Cell.

[62] Matsuzawa-Ishimoto Y., Shono Y., Gomez L.E., Hubbard-Lucey V.M., Cammer M., Neil J., Dewan M.Z., Lieberman S.R., Lazrak A., Marinis J.M. (2017). Autophagy protein ATG16L1 prevents necroptosis in the intestinal epithelium. J. Exp. Med..

[63] Pott J., Kabat A.M., Maloy K.J. (2018). Intestinal epithelial cell autophagy is required to protect against TNF-induced apoptosis during chronic colitis in mice. Cell Host Microbe.

[64] Cadwell K., Liu J.Y., Brown S.L., Miyoshi H., Loh J., Lennerz J.K., Kishi C., Kc W., Carrero J.A., Hunt S. (2008). A key role for autophagy and the autophagy gene Atg16l1 in mouse and human intestinal Paneth cells. Nature.

[65] Xiao L., Rao J.N., Wang J.Y. (2021). RNA-binding proteins and long noncoding RNAs in intestinal epithelial autophagy and barrier function. Tissue Barriers.

[66] Xiao L., Rao J.N., Cao S., Liu L., Chung H.K., Zhang Y., Zhang J., Liu Y., Gorospe M., Wang J.Y. (2016). Long noncoding RNA SPRY4-IT1 regulates intestinal epithelial barrier function by modulating the expression levels of tight junction proteins. Mol. Biol. Cell.

[67] Xiao L., Wang J.Y. (2014). RNA-binding proteins and microRNAs in gastrointestinal epithelial homeostasis and diseases. Curr. Opin. Pharmacol..

[68] Wang J.Y., Xiao L., Wang J.Y. (2017). Posttranscriptional regulation of intestinal epithelial integrity by noncoding RNAs. Wiley Interdiscip. Rev. RNA.

[69] Zou T., Jaladanki S.K., Liu L., Xiao L., Chung H.K., Wang J.Y., Xu Y., Gorospe M., Wang J.Y. (2016). H19 long noncoding RNA regulates intestinal epithelial barrier function via microRNA 675 by interacting with RNA-binding protein HuR. Mol. Cell. Biol..

[70] Cao S., Xiao L., Rao J.N., Zou T., Liu L., Zhang D., Turner D.J., Gorospe M., Wang J.Y. (2014). Inhibition of Smurf2 translation by miR-322/503 modulates TGF-beta/Smad2 signaling and intestinal epithelial homeostasis. Mol. Biol. Cell.

[71] Salmena L., Poliseno L., Tay Y., Kats L., Pandolfi P.P. (2011). A ceRNA hypothesis: The Rosetta Stone of a hidden RNA language?. Cell.

[72] Thomson D.W., Dinger M.E. (2016). Endogenous microRNA sponges: Evidence and controversy. Nat. Rev. Genet..

[73] Xiao L., Rao J.N., Zou T., Liu L., Cao S., Martindale J.L., Su W., Chung H.K., Gorospe M., Wang J.Y. (2013). miR-29b represses intestinal mucosal growth by inhibiting translation of cyclin-dependent kinase 2. Mol. Biol. Cell.

[74] Wang J.Y., Cui Y.H., Xiao L., Chung H.K., Zhang Y., Rao J.N., Gorospe M., Wang J.Y. (2018). Regulation of intestinal epithelial barrier function by long noncoding RNA uc.173 through interaction with microRNA 29b. Mol. Cell. Biol..

[75] Xiao L., Wu J., Wang J.Y., Chung H.K., Kalakonda S., Rao J.N., Gorospe M., Wang J.Y. (2018). Long noncoding RNA *uc. 173* promotes renewal of the intestinal mucosa by inducing degradation of microRNA 195. Gastroenterology.

[76] Kwon M.S., Chung H.K., Xiao L., Yu T.X., Wang S.R., Piao J.J., Rao J.N., Gorospe M., Wang J.Y. (2021). MicroRNA-195 regulates Tuft cell function in the intestinal epithelium by altering translation of DCLK1. Am. J. Physiol. Cell Physiol..

[77] Zhang Y., Zhang Y., Xiao L., Yu T.X., Li J.Z., Rao J.N., Turner D.J., Gorospe M., Wang J.Y. (2017). Cooperative repression of insulin-like growth factor type 2 receptor translation by microRNA 195 and RNA-binding protein CUGBP1. Mol. Cell. Biol..

[78] Glisovic T., Bachorik J.L., Yong J., Dreyfuss G. (2008). RNA-binding proteins and post-transcriptional gene regulation. FEBS Lett..

[79] Keene J.D. (2007). RNA regulons: Coordination of post-transcriptional events. Nat. Rev. Genet..

[80] Brennan C.M., Steitz J.A. (2001). HuR and mRNA stability. Cell. Mol. Life Sci..

[81] Brennan S.E., Kuwano Y., Alkharouf N., Blackshear P.J., Gorospe M., Wilson G.M. (2009). The mRNA-destabilizing protein tristetraprolin is suppressed in many cancers, altering tumorigenic phenotypes and patient prognosis. Cancer Res..

[82] Kelaini S., Chan C., Cornelius V.A., Margariti A. (2021). RNA-binding proteins hold key roles in function, dysfunction, and disease. Biology.

[83] Gebauer F., Schwarzl T., Valcarcel J., Hentze M.W. (2021). RNA-binding proteins in human genetic disease. Nat. Rev. Genet..

[84] Chung H.K., Xiao L., Jaladanki K.C., Wang J.Y. (2021). Regulation of Paneth cell function by RNA-binding proteins and noncoding RNAs. Cells.

[85] Sharma S., Xiao L., Wang J.Y. (2023). HuR and its interactions with noncoding RNAs in gut epithelium homeostasis and diseases. Front. Biosci. (Landmark Ed.).

[86] Liu L., Xiao L., Chung H.K., Kwon M.S., Li X.X., Wu N., Rao J.N., Wang J.Y. (2019). RNA-binding protein HuR regulates Rac1 nucleocytoplasmic shuttling through nucleophosmin in the intestinal epithelium. Cell. Mol. Gastroenterol. Hepatol..

[87] Du W.W., Zhang C., Yang W., Yong T., Awan F.M., Yang B.B. (2017). Identifying and characterizing circRNA-Protein interaction. Theranostics.

[88] Pamudurti N.R., Bartok O., Jens M., Ashwal-Fluss R., Stottmeister C., Ruhe L., Hanan M., Wyler E., Perez-Hernandez D., Ramberger E. (2017). Translation of circRNAs. Mol. Cell.

[89] Yang Y., Fan X., Mao M., Song X., Wu P., Zhang Y., Jin Y., Yang Y., Chen L.L., Wang Y. (2017). Extensive translation of circular RNAs driven by *N*^6^-methyladenosine. Cell Res..

[90] Ananthakrishnan A.N., Kaplan G.G., Ng S.C. (2020). Changing global epidemiology of inflammatory bowel diseases: Sustaining health care delivery into the 21st century. Clin. Gastroenterol. Hepatol..

[91] Kaplan G.G. (2015). The global burden of IBD: From 2015 to 2025. Nat. Rev. Gastroenterol. Hepatol..

[92] Lun J., Guo J., Yu M., Zhang H., Fang J. (2023). Circular RNAs in inflammatory bowel disease. Front. Immunol..

[93] Qiao Y.Q., Cai C.W., Shen J., Zheng Q., Ran Z.H. (2019). Circular RNA expression alterations in colon tissues of Crohn’s disease patients. Mol. Med. Rep..

[94] Yin J., Hu T., Xu L., Li P., Li M., Ye Y., Pang Z. (2019). Circular RNA expression profile in peripheral blood mononuclear cells from Crohn disease patients. Medicine.

[95] Li B., Li Y., Li L., Yu Y., Gu X., Liu C., Long X., Yu Y., Zuo X. (2021). Hsa_circ_0001021 regulates intestinal epithelial barrier function via sponging miR-224-5p in ulcerative colitis. Epigenomics.

[96] Bala M., Catena F., Kashuk J., De Simone B., Gomes C.A., Weber D., Sartelli M., Coccolini F., Kluger Y., Abu-Zidan F.M. (2022). Acute mesenteric ischemia: Updated guidelines of the World Society of Emergency Surgery. World J. Emerg. Surg..

[97] Gonzalez L.M., Moeser A.J., Blikslager A.T. (2015). Animal models of ischemia-reperfusion-induced intestinal injury: Progress and promise for translational research. Am. J. Physiol.-Gastrointest. Liver Physiol..

[98] Qi X., Zhang D.H., Wu N., Xiao J.H., Wang X., Ma W. (2015). ceRNA in cancer: Possible functions and clinical implications. J. Med. Genet..

[99] Chen L.L., Yang L. (2015). Regulation of circRNA biogenesis. RNA Biol..

[100] Vo J.N., Cieslik M., Zhang Y., Shukla S., Xiao L., Zhang Y., Wu Y.M., Dhanasekaran S.M., Engelke C.G., Cao X. (2019). The landscape of circular RNA in cancer. Cell.

[101] Li Y., Zheng Q., Bao C., Li S., Guo W., Zhao J., Chen D., Gu J., He X., Huang S. (2015). Circular RNA is enriched and stable in exosomes: A promising biomarker for cancer diagnosis. Cell Res..

[102] Jia G., Li Y., Yuan S., Li X., Chen L., Zhang S., Li M., Xu H., Liang H. (2025). Multiple regulatory events contribute to a widespread circular RNA downregulation in precancer and early stage of colorectal cancer development. Biomark. Res..

[103] Dou Y., Cha D.J., Franklin J.L., Higginbotham J.N., Beckler A.D., Weaver A.M., Prasad K., Levy S., Coffey R.J., Patton J.G. (2016). Circular RNAs are down-regulated in KRAS mutant colon cancer cells and can be transferred to exosomes. Sci. Rep..

[104] Zhao S., Zheng X., Chen S., Liang X., Zhang L., Liu Y., Wang J., Ma L. (2023). Characterization of cell-type specific circular RNAs associated with colorectal cancer metastasis. NAR Cancer.

[105] Huang A., Zheng H., Wu Z., Chen M., Huang Y. (2020). Circular RNA-protein interactions: Functions, mechanisms, and identification. Theranostics.

[106] Kristensen L.S., Jakobsen T., Hager H., Kjems J. (2022). The emerging roles of circRNAs in cancer and oncology. Nat. Rev. Clin. Oncol..

[107] Hansen T.B. (2018). Improved circRNA Identification by Combining Prediction Algorithms. Front. Cell Dev. Biol..

[108] Digby B., Finn S., Ó Broin P. (2024). Computational approaches and challenges in the analysis of circRNA data. BMC Genom..

[109] Vromman M., Vandesompele J., Volders P.J. (2021). Closing the circle: Current state and perspectives of circular RNA databases. Brief. Bioinform..

